# Developing and validating a machine learning prognostic model for alerting to imminent deterioration of hospitalized patients with COVID-19

**DOI:** 10.1038/s41598-022-23553-7

**Published:** 2022-11-10

**Authors:** Yuri Kogan, Ari Robinson, Edward Itelman, Yeonatan Bar-Nur, Daniel Jorge Jakobson, Gad Segal, Zvia Agur

**Affiliations:** 1 Institute for Medical Biomathematics (IMBM), Hate’ena St., 10, P.O. Box 282, 6099100 Bene Ataroth, Israel; 2 Department of Internal Medicine I, Chaim Sheba Medical Center, Sackler Faculty of Medicine, Tel Aviv University, Tel Aviv, Israel; 3 Intensive Care Unit, Barzilai University Medical Center, 78278 Ashkelon, Israel; 4 Faculty of Health Sciences, Ben-Gurion University of the Negev, Beer-Sheba, Israel

**Keywords:** Prognosis, Infectious diseases, Viral infection, Health care, Computational biology and bioinformatics, Machine learning, Predictive medicine

## Abstract

Our study was aimed at developing and validating a new approach, embodied in a machine learning-based model, for sequentially monitoring hospitalized COVID-19 patients and directing professional attention to patients whose deterioration is imminent. Model development employed real-world patient data (598 prediction events for 210 patients), internal validation (315 prediction events for 97 patients), and external validation (1373 prediction events for 307 patients). Results show significant divergence in longitudinal values of eight routinely collected blood parameters appearing several days before deterioration. Our model uses these signals to predict the personal likelihood of transition from non-severe to severe status within well-specified short time windows. Internal validation of the model's prediction accuracy showed ROC AUC of 0.8 and 0.79 for prediction scopes of 48 or 96 h, respectively; external validation showed ROC AUC of 0.7 and 0.73 for the same prediction scopes. Results indicate the feasibility of predicting the forthcoming deterioration of non-severe COVID-19 patients by eight routinely collected blood parameters, including neutrophil, lymphocyte, monocyte, and platelets counts, neutrophil-to-lymphocyte ratio, CRP, LDH, and D-dimer. A prospective clinical study and an impact assessment will allow implementation of this model in the clinic to improve care, streamline resources and ease hospital burden by timely focusing the medical attention on potentially deteriorating patients.

## Introduction

Over the past two years, many countries have witnessed their healthcare systems being overwhelmed by patients infected with the SARS-CoV-2 virus, which causes the pandemic known as COVID-19. The heavy hospital load during the pandemic carries multiple risks for patients and causes serious concern worldwide^1–3^. In Israel, the in-hospital mortality rate of patients with COVID-19 significantly increased during a moderately heavy patient load; excess mortality is associated with rapid escalation in the number of hospitalized patients carrying the disease^3^. Similar results were obtained in hospitals in the USA^4^, Greece^5^, and other countries.

The recent outbreaks of the fast-spreading variant, B.1.1.529 (Omicron), and related subsequent variants, caused an additional hospital burden, as these variants debilitated large proportions of healthcare workers and caused critical staffing shortages in hospitals. It is not unlikely that such outbreaks will be witnessed in future waves of COVID-19 worldwide. One way to increase the quality of care and attain better clinical outcomes in these circumstances is to focus the healthcare professionals' attention on patients having a high risk of imminent deterioration while easing the tight surveillance of other mild and moderate patients.

Aiming to tackle the problem of overburdened healthcare systems under a high COVID-19 caseload, we developed a prediction model, purported to be employed consecutively throughout the patient’s stay in the hospital, alerting the physicians when anticipating deterioration within two or four days. The model uses the power of machine learning (ML) to extract evolving deterioration signals from routine, longitudinally collected blood tests and processes them for computing the likelihood of the patient's deterioration within the next 48 or 96 h.

Most prognostic models for COVID-19 aim to provide an early-stage triage and are applied only once for a patient^6,7^, thus limiting their applicability^8–10^. In contrast, our model is aimed at consecutively monitoring hospitalized patients, as of the third day after admission, predicting the risk of deterioration during a narrow time window. The patients' dataset, selected for the development and the testing work, included this target population (see inclusion criteria in the “Methods” section).

The electronic health records (EHRs) of the patients in our target population were selected from all patients treated at Sheba Medical Center, Israel (to be denoted Sheba), from March 2020 to August 2021 (a total of 803 patients; see Table 1). The Sheba dataset included blood test results, and daily patient statuses, evaluated by the physicians, based upon internationally accepted guidelines^11^, with local adaptations by the Israeli Ministry of Health^12^ (see details in “Methods”). This dataset was divided into two subsets: one for the training of the model and one for its internal validation. External validation was made using a clinical dataset of COVID-19 patients staying at Barzilai University Medical Center, Israel (to be denoted Barzilai), during the same period (Table 1).

**Table 1:**
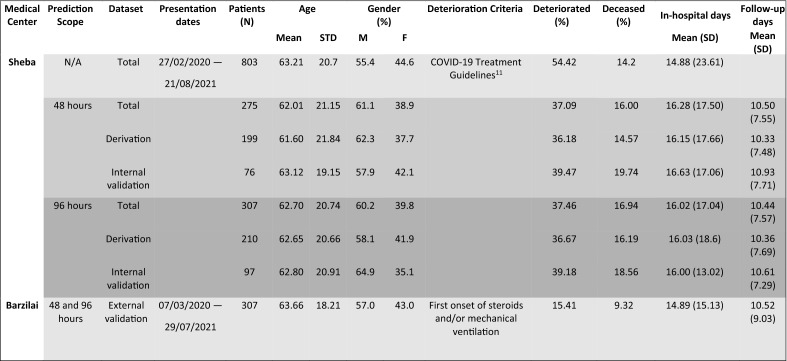
Demographic data, patient characteristics, and division into study arms of patients collected in Sheba Medical Center and Barzilai University Medical Center.

## Results

### Divergence of blood parameters before deterioration

**Figure 1:**
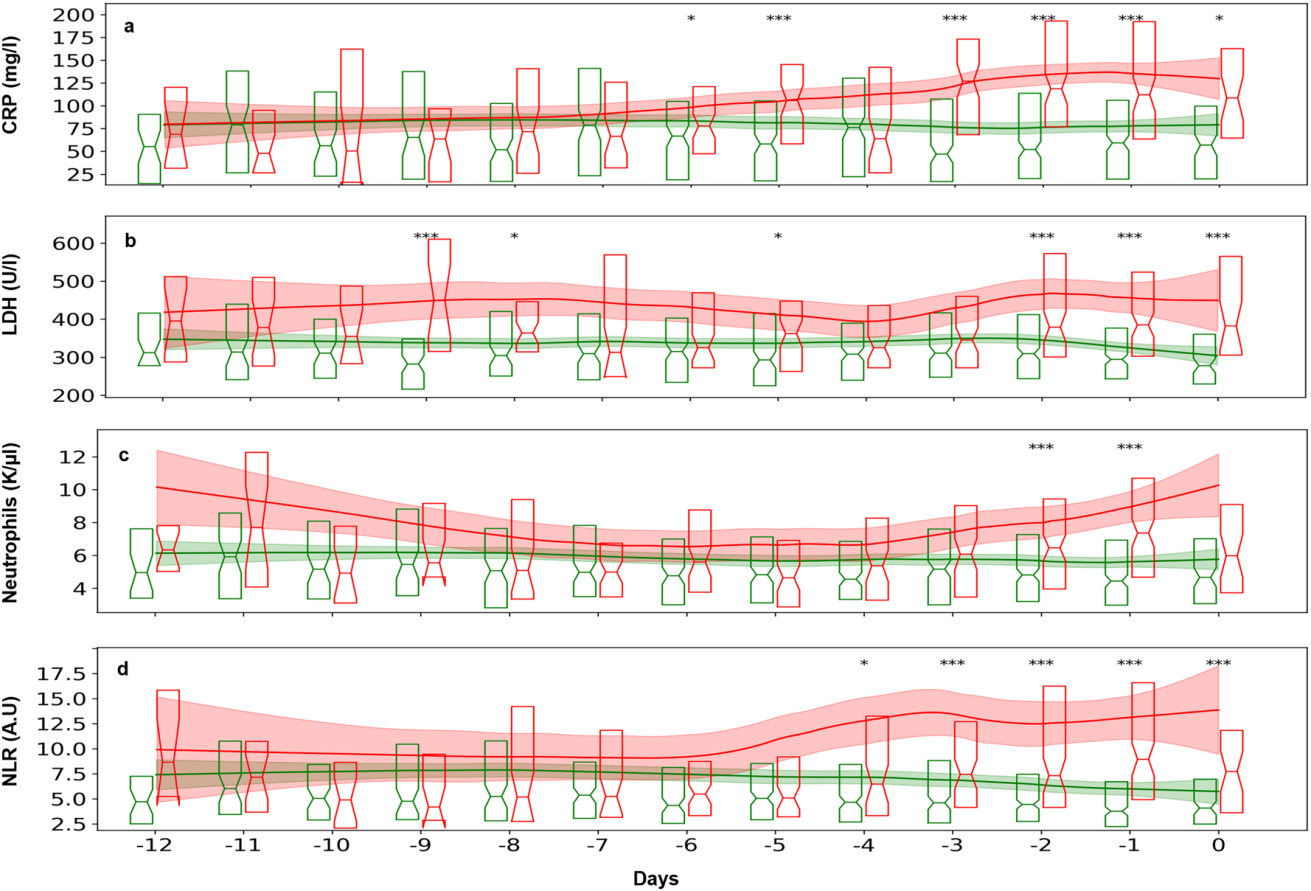
Longitudinal measurements of blood parameters in mild and moderate patients who would deteriorate and in those who would not deteriorate. Patients with COVID-19, collected in Sheba Medical Center, were aligned to t = 0 by the day of their first deterioration from a mild or moderate status to a severe status (red), or by the end of follow-up for patients who did not deteriorate (green). Summary boxplots, show 25th, 50th, and 75th percentiles of pooled daily (**a**) CRP values of 581 continually non-severe and 207 potentially severe patients, (**b**) LDH values of 591 non-severe and 206 severe patients, (**c**) absolute neutrophil counts of 595 non-severe and 208 severe patients, (**d**) NLR values of 595 non-severe and 208 severe patients. Locally Weighted Scatterplot Smoothing (lowess), with 95% confidence intervals are shown in continuous lines and light bands around them. Asterisks represent the level of significance of the differences between the daily status groups, determined by the p-values of the Kruskall-Wallis test, *< 0.05, **< 0.01, ***< 0.001. The test for difference was applied to the values collected at each day, separately (e.g., test whether the values between − 24 h and 0 are different, test whether the values between − 48 and − 24 h are different, etc.).

Eight hundred and three patients from Sheba were selected for the initial comparison of the longitudinal blood parameter values, using our inclusion criteria set 1 (see “Methods”); 208 of these 803 patients have deteriorated and 595 have not. Significant divergence in blood parameters between *non-severe* and *severe* patients was found in the week preceding the deterioration of the latter (see “Methods” for the definition of *non-severe* and *severe* patients). This was so for C-reactive protein (CRP), Lactate dehydrogenase (LDH), neutrophil count, and the ratio of neutrophil to lymphocyte counts (NLR; Fig. 1). Additional blood parameters demonstrated similar but somewhat less significant, divergence (lymphocytes, monocytes, platelets, D-dimer; see Supplementary Information, Fig. S1).

Inspecting Fig. 1 and Fig. S1, one sees that the values of blood parameters in non-deteriorating patients remained roughly stable throughout hospitalization. A similar stability is manifested for the early values of blood parameters in the deteriorating patients until about one week before deterioration, after which they change significantly. This observation led us to hypothesize that the changing dynamics of blood parameters, which begins several days before breakdown, can be used as a signal heralding the individual patient's deterioration. Based on these results, we further hypothesized that one could develop a model based on a combination of the longitudinal measurements of the above parameters, to predict deterioration of currently *non-severe* patients before it becomes clinically apparent. These hypotheses were tested, as is described below.

### Model training

The dataset collected at Sheba and selected to represent the target population for our model, according to the inclusion criteria set 2, comprised 913 prediction time points (denoted events) in 307 patients for the prediction scope (window) of 96 h, and 813 events of 275 patients for the prediction scope of 48 h. After the training/testing split, the training sets included 210 and 199 patients, with 598 and 563 events, and the testing sets included 97 and 76 patients, with 315 and 250 events for the prediction scopes of 96 h and 48 h, respectively. The entire training procedure was carried out on the training sets, and the testing sets were kept aside for internal validation.

**Figure 2:**
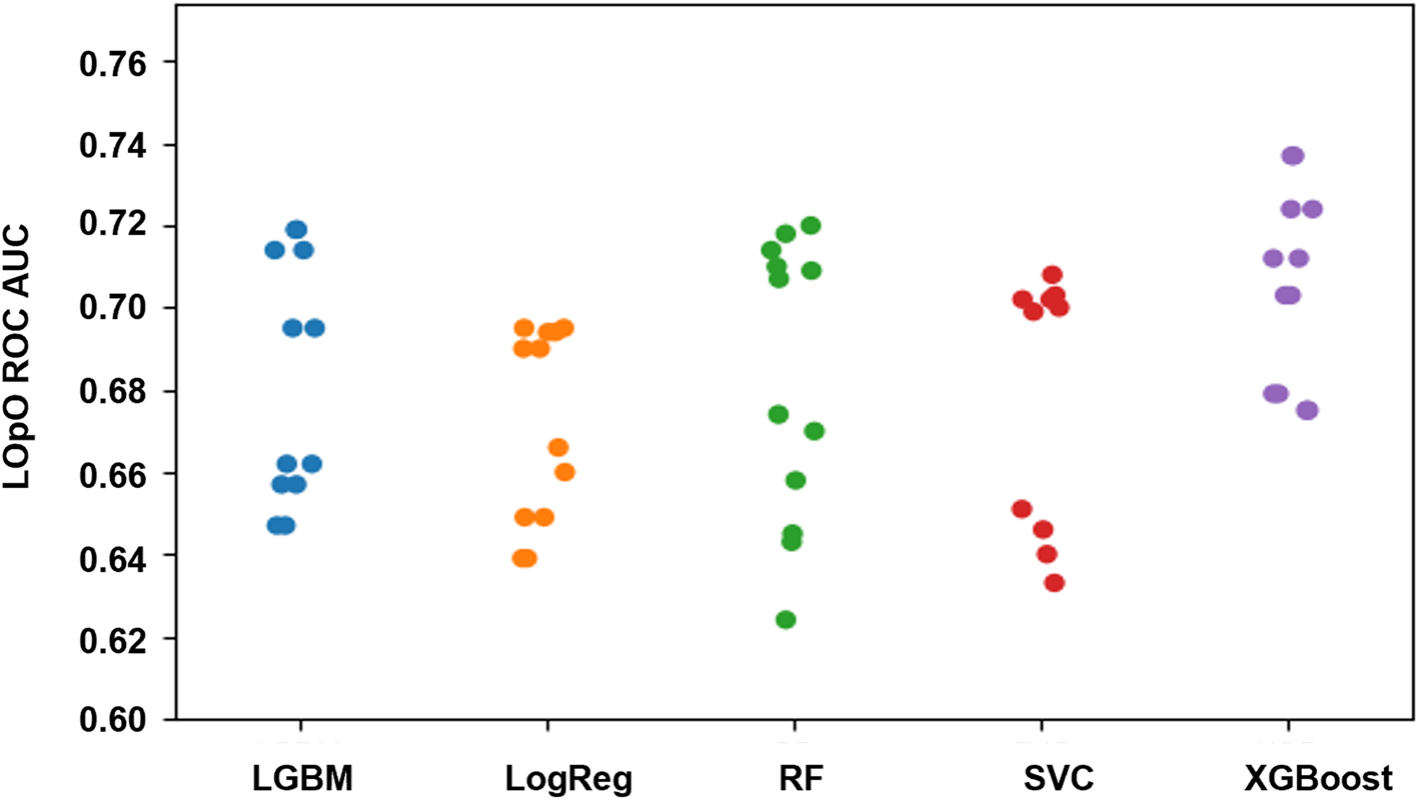
Selection of the ML model. The model performance was estimated by Leave-one-patient-out cross-validation (LOpO CV) on the training set of the Sheba Medical Center cohort, comparing of the ROC AUC of 5 different ML models. Each dot stands for (1) a specific ML model, as marked on the abscissa, (2) a specific prediction scope (48 h or 96 h), and (3) a specific choice of the set of dynamic features. The density and location of the cloud, formed by the aggregation of dots for each ML model indicate the model robustness and accuracy.

During the training, the robustness of the performance of different ML models was examined across various sets of dynamic features, tuning grids, and weights assigned to the *non-severe* and *severe* outcomes. Figure 2 displays the results, showing the models’ performance, measured by ROC AUC, as evaluated by Leave One Patient Out Cross Validation (LOpO CV) on the training set for the two prediction scopes (48 h or 96 h). For each model the location and spread of the dots indicate the prediction accuracy and the robustness, respectively. In Fig. 2, one can see that the XGBoost model provides favorable and robust results, as compared to other models. A reduction of different input blood parameters, from eight to six or to four, was also attempted, resulting in a significant decrease in accuracy (see Supplementary Information, Fig. S2).

### Internal validation by an independent testing subset of the Sheba cohort

**Figure 3:**
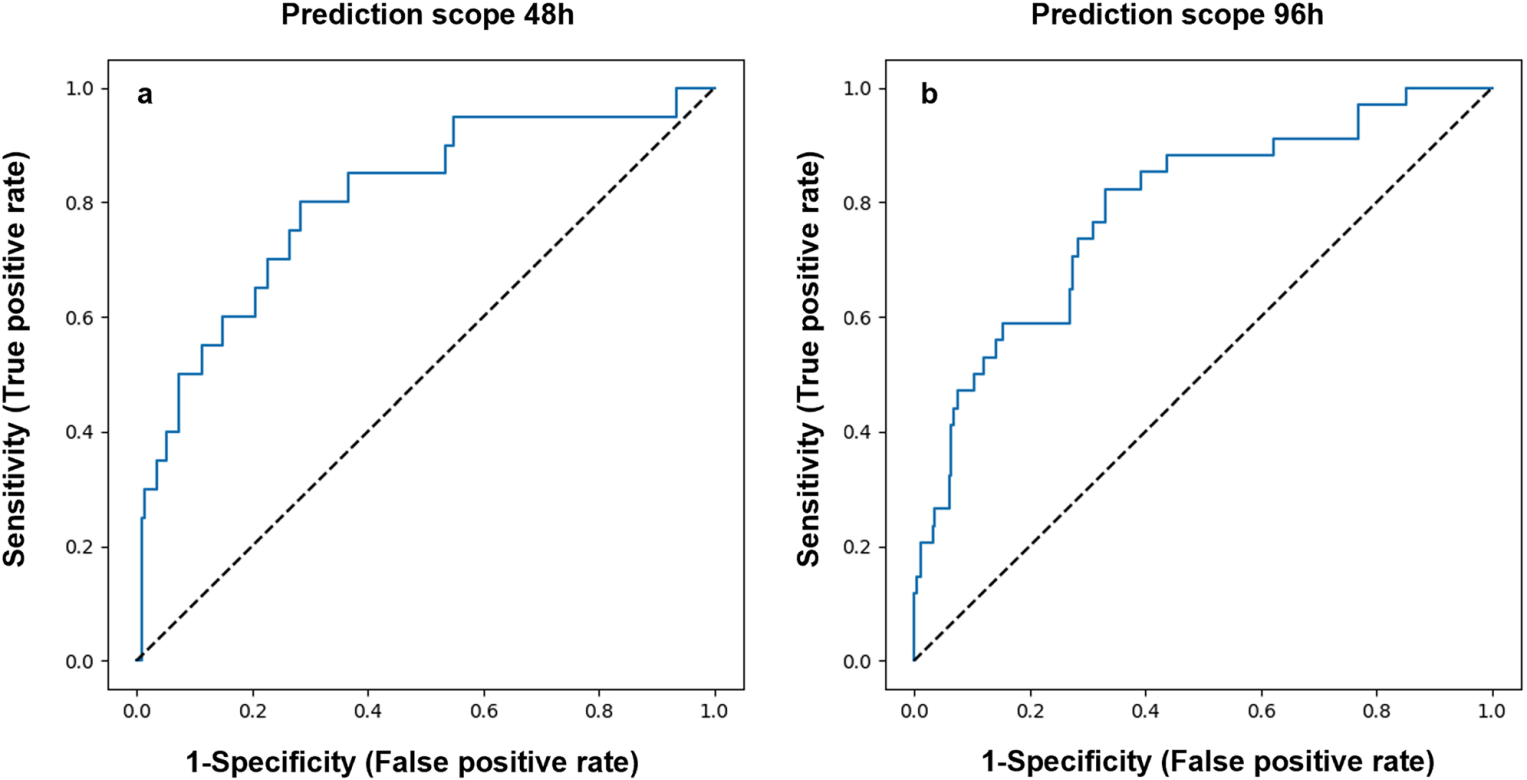
Internal validation results. Model accuracy, measured on the internal validation dataset from Sheba Medical Center, as shown in ROC curves, for prediction scopes of (**a**) 48 h and (**b**) 96 h.

The prediction capacity of the chosen XGBoost model was evaluated on the internal validation sets of Sheba patients. The resulting ROC curves had AUC of 0.8 (95% CI 0.75–0.86) and 0.79 (95% CI 0.74–0.83) for predicting deterioration within 48 or 96 h, respectively (Fig. 3a,b). Applying this model with the classification threshold tuned for the objective of maximal specificity, constrained by at least 60% sensitivity, results in a specificity of 60.9% (95% CI 57.6–64%) and 73% (95% CI 70.3–75.8%) and sensitivity of 85% (95% CI 77–93.5%) and 61.8% (95% CI 53.1–70.1%), for the prediction scopes of 48 and 96 h, respectively. Applying the model with the threshold of 0.5 resulted in a specificity of 91.7% (95% CI 89.9–93.5%) and 90.7% (95% CI 89–92.5%), and sensitivity of 50% (95% CI 38.1–61.3%), and 47.1% (95% CI 38.1–55.4%), for the prediction windows of 48 h and 96 h, respectively.

### Validation by a cohort from a different hospital

**Figure 4:**
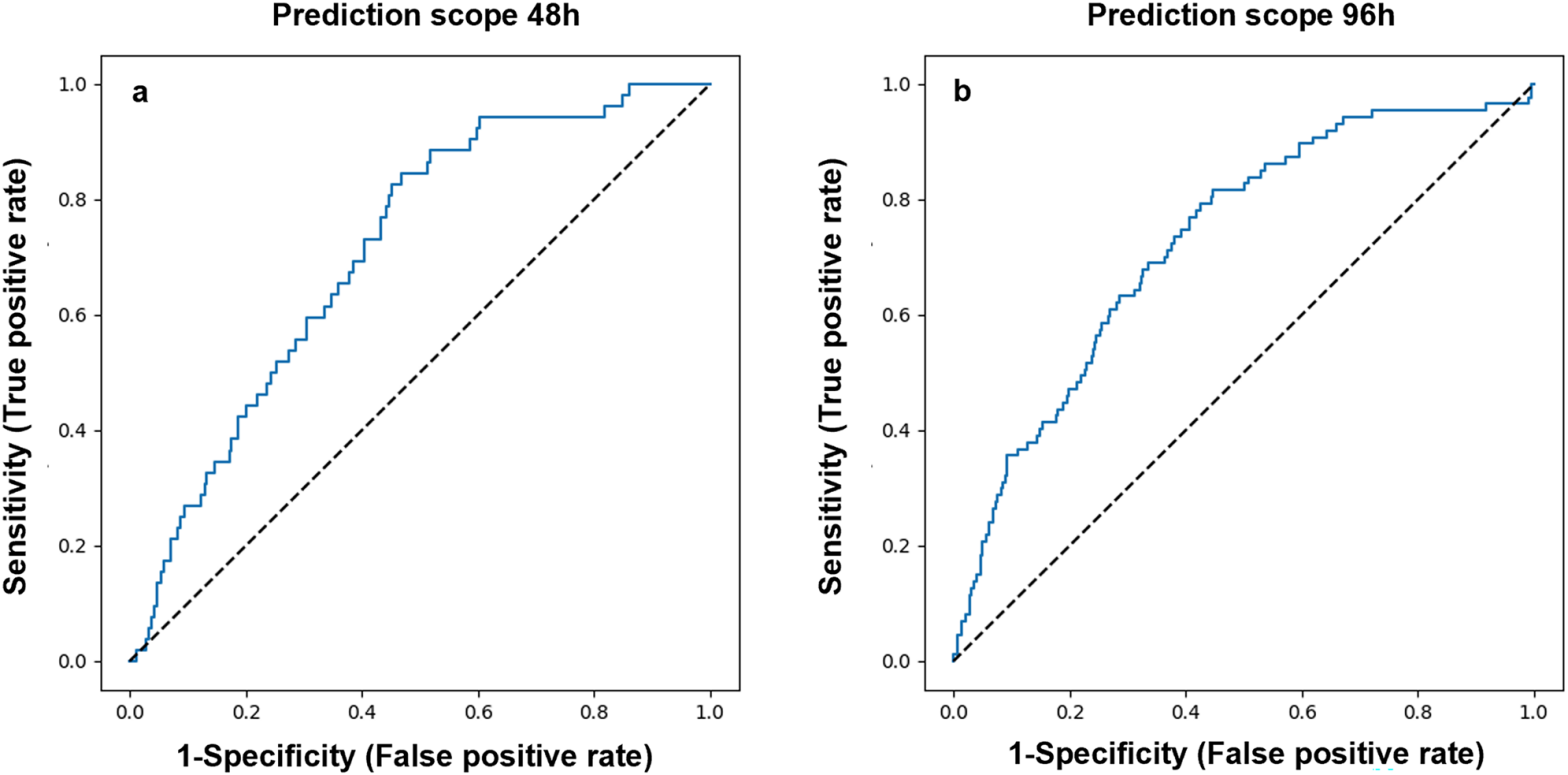
External validation results. Model accuracy, measured on the external validation dataset from Barzilai University Medical Center, as shown in the ROC curves, for prediction scopes of (**a**) 48 h and (**b**) 96 h.

For the external model validation, the EHRs of 279 patients (1328 events) were used, selected by our inclusion criteria set 3, from the Barzilai cohort (see “Methods”). Model application, starting 2 days after hospital admission, with its threshold tuned to a target of maximal specificity with at least 60% sensitivity, resulted in ROC AUC of 0.7 (95% CI 0.67–0.73) and 0.72 (95% CI 0.69–0.75), specificity of 64.7% (95% CI 63.3–65.9%) and 65.3% (95% CI 64.1–66.7%), and sensitivity of 60.3% (95% CI 53.3–66.8%) and 65.9% (95% CI 60.8–71.5%), for the prediction scope of 48 and 96 h, respectively (Fig. 4a,b).

## Discussion

Due to the high pathogenicity of the SARS-CoV-2, B.1.1.529 variant (Omicron), its recent outbreak ramped up wildly, creating an excessive demand on hospital beds and equipment, and low staffing levels, as front-line workers were infected with this contagious variant. As outbreaks of new variants in the future can lead to similar crises, our aim was to develop a prediction model as the basis of a clinical alert tool intended to ease the pressure in hospitals due to increased numbers of patients requiring care and dwindling resources.

Our target population includes all *non-severe* patients with COVID-19, hospitalized for a sufficiently long time (at least 48 h) and having routinely collected daily records of blood tests. The model is aimed to identify patients who approach deterioration by following up *non-severe* patients from the third hospitalization day until deterioration or discharge. By predicting upcoming deterioration, the model will enable the physicians to optimally time the decision about a more targeted treatment for this patient, e.g., by corticosteroids^13–17^. With the ongoing patient stratification this model is expected to offer, it may also serve for streamlining resource allocation in times of stress, especially when efficacious yet costly anti-viral medications become available.

Good model performance was demonstrated on the internal validation dataset from Sheba, and moderate performance was demonstrated on the external validation dataset from a different hospital (Barzilai) located in another region of the country. One would expect a lower prediction accuracy in the Barzilai cohort than in the Sheba cohort, as is usually the case when validating a predictor on data from a different source and given the differences between the two hospitals in how deterioration could be evaluated retrospectively. Yet, the moderately good accuracy in predicting deterioration events, which were differently evaluated in the two cohorts, pinpoints the model resilience, and substantiates its further development and examination in other patient populations. The performance is equally good on patients harboring a variety of SARS-CoV-2, as attested by the similarity of the prediction accuracy across different periods of the pandemic until August 2021. The prediction accuracy of a potential deterioration within 48 h is comparable to that of deterioration within 96 h, and it remains for the physician to decide on the more clinically useful prediction scope.

The model's specificity and sensitivity guarantee that most of the deterioration events will be flagged as such during the four preceding days, whereas most of the no deterioration events will not be flagged as deterioration in the 4 former days. A different choice of classification threshold was also considered, to ascertain the model's specificity rather than its sensitivity. Setting the model's sensitivity threshold value to 0.5, a high specificity was obtained, assuring that almost all events of no clinical deterioration will not be signaled as potential deterioration. However, this comes at the cost of relatively low sensitivity to really deteriorating patients (Table S1).

Hond et al.^18^ systematically review published guidelines for developing, evaluating, and implementing artificial intelligence-based prediction models in healthcare. They provide an exhaustive list of aspects and issues to address during the six developmental phases of prognostic models. The present article reports the first three phases of the process (data preparation, model development, and model validation). While meeting most of their recommendations for these phases, which pertain to the present context, some challenges and limitations remain, as is detailed below.

In our work, the inclusion criteria applied for selecting patients from the database were only those derived from the definition of the target population for which the model is purported, including the requirement for routine blood test results. Note that the model can work when some of the measurements are missing, due to imputation methods. Our derivation and validation datasets did not make multiple uses of the same data, nor did they include inappropriate data sources. During the training, the performance of five ML models was compared across plural setups of precisely defined dynamic features. Model tuning, feature selection, and the final model selection were performed by cross-validation (CV) on the training set, and the final model was validated internally and externally. Overestimation of the model accuracy can occur due to potential correlations between the data points of the same patient. This was prevented by grouping events by patient in the training/testing split. It is our belief that our attentiveness in model development and validation prevented most of the pitfalls identified in previous prediction models.

The main limitation of our work pertains to the derivation of the data from two clinical centers in the same country, collected over a relatively short period. This limitation may create a representation bias, due to differences in the targeted patient populations and in the pertinent clinical practices in different parts of the world. Note also that there is an inherent outcome imbalance due to the unique nature of our prediction task: multiple non-deterioration events, but only one deterioration event, at the most, can be associated with each patient. This was mitigated by including different class weighting schemes in tuning the hyper-parameters, but this imbalance still affects the classification metrics. We suggest adapting the metrics for evaluating model performance when monitoring the patients consecutively to anticipate a critical clinical event. It is also required to address the feasibility, practicality, and benefit in integration of the model in the clinical workflow by a prospective clinical trial, yet to be performed.

The work presented here describes one of the first COVID-19 prediction models that may enable daily evaluation of the patient’s deterioration likelihood. This is achieved by a unique approach to the analysis of dynamic changes in the standard blood test data, using trends in changes in blood counts or biochemical parameters as features in the ML analysis. The model, requiring a daily input of only eight, routinely collected, blood parameters, enables prediction of deterioration within the coming 48 or 96 h, when an effective change in treatment can be made sufficiently early to arrest the decline. Noy et al.^19^ developed a comparable model predicting patients’ deterioration in the next 7–30 h, as evaluated by the modified NEWS2 score and using an hourly input of an extensive panel of demographic and clinical parameters. Note that the pragmatism motivating our choice of a minimal set of input parameters is traded off by the somewhat reduced accuracy of our model, as compared to Noy et al.^19^.

Previous studies use blood parameter values and age, or gender, to predict severity or death in patients with COVID-19. Shang and colleagues^20^ suggest that NLR, CRP, and platelets, evaluated upon hospital admission, can effectively be used to predict disease severity. Another study reports a striking distinction in all early measurements of neutrophils, lymphocytes, high-sensitivity CRP, and LDH, between patients who eventually suffered fatal outcomes and those who survived^21^. In contrast, our work shows that it is only a few days before deterioration, that routine blood parameters of potentially severe patients, namely, CRP, LDH, neutrophils, lymphocytes, NLR, monocytes, platelets, and D-dimer, diverge from those of persistently mild or moderate patients.

Numerous prognostic models have been developed for COVID-19, using both traditional statistics and ML. Blood parameters, like ferritin^22^, troponin, and myoglobin^23–26^, demographic parameters, including Charlson comorbidity index score^27^, age and gender^28,29^, chest CT images^30^, routine chest X-rays^31^, age-related dementia^32^, cardiac auscultation^33^ and other factors are suggested to predict severity or death in patients with COVID-19. However, all these studies were aimed at a single-time assessment of patients’ prognosis, close to admission. Note, that grave pitfalls were identified in most of the published prediction models^9,10^.

The work presented here differs from other prognostic models in its primary goal that, necessarily, limits the repertoire of potential predictors used by the model. Since this work aimed to create a supportive tool for the physicians, which would signal an imminent deterioration of hospitalized patients with COVID-19, the tool must be *practical* and *applicable* in hospitals worldwide.

The practicality prerequisite has guided our objective to limit the number of input predictors in the final model to the minimal number that can jointly yield accurate predictions. Therefore, all the routinely evaluated blood parameters were tested, but those that, even though they may have correlated with disease progression, did not significantly contribute to the prediction accuracy of the model, were excluded from the input predictor list. Other predictors, suggested in the literature to correlate with disease severity, are not measured routinely in many COVID-19 wards. For example, in Sheba or Barzilai datasets, ferritin, myoglobin and troponin are only sporadically measured and other suggested predictors, including age, sex and comorbidities, are not reported at all. These parameters were not included in the study, a priori.

Moreover, for daily evaluation of the patient's prognosis, the potential predictors, whose values "feed" the model, must be *dynamic* within a time scale of days/weeks and be measured sufficiently *frequently*. For example, the age of the patients—negligibly changing during hospitalization—cannot be strongly informative for the question, “will the patient deteriorate in the coming 48/96 h?" All these prerequisites determined our decision to study only clinical parameters that are evaluated and registered daily or at least three times weekly in all or most hospitalized patients with COVID-19.

Employing only eight routinely evaluated blood parameters, it was shown that one can predict imminent patient deterioration already after two days following admission to the hospital when at least two blood samples are available. Yet, to render our model applicable in the clinic, it must be completed by an observatory prospective clinical study. Such a study will check if the model is sufficiently accurate to capture the timing of patient deterioration, if its implementation in the clinical setting is possible, and if focusing care on patients, having high-risk for imminent deterioration, can aid in alleviating the burden of excessive hospital load of patients with COVID-19.

We foresee a vital role for dynamic prediction models as a potential adaptive, prognostic tool for physicians and administrators alike. Such models can be also used in ambulatory care under remote medicine and frequent blood tests. Since the severity of the disease inflicted by future SARS-CoV-2 variants is expected to still depend upon the activation of the patient’s immune system and since our model relies mainly on immune-related variables, it will likely also be effective for future SARS-CoV-2 variants.

## Methods

### Clinical data

The study was reviewed and approved by the Sheba Medical Center Institutional Review Board (IRB)-Helsinki Committee (7953-20-SMC), and by the Barzilai University Medical Center IRB (BRZ-0003-21) and conformed to the principles outlined in the declaration of Helsinki. All methodologies were performed according to the relevant guidelines and regulations, and patient data were anonymized. The Sheba Medical Center IRB-Helsinki Committee and the Barzilai University IRB/Ethics (Helsinki) Committee approved the waiver of informed consent. Out of all the EHRs of the patients hospitalized in all wards of Sheba and Barzilai between March 2020 and August 2021, those who met our inclusion criteria were extracted (see subsection below). From the anonymized EHRs, the longitudinal measurements of eight blood parameters, namely, the whole blood counts in a microliter of lymphocytes, neutrophils, monocytes, and platelets, the NLR, and the serum concentrations or activity of CRP, LDH, D-Dimer, were collected. The longitudinal clinical status evaluations (Sheba), and the dates of commencing steroid therapy and mechanical ventilation (Barzilai) were extracted for daily evaluation of the patient's status.

### Inclusion criteria

Three sets of inclusion criteria, adjusted for the analysis and the structure of the datasets, were employed:

- Inclusion criteria set 1: For the initial comparison of the longitudinal values of blood parameters between the non-deteriorating and deteriorating patients, the patients from Sheba were included if they (1) had an initial *non-severe* status evaluation, (2) had at least two days follow-up before becoming *severe* (deterioration) or discharge from hospital without deterioration, and (3) had at least one blood test record in the two weeks preceding the end of the follow-up.
- Inclusion criteria set 2: For the development and internal validation of the model, the patients from Sheba were included if they (1) had an initial *non-severe* status evaluation, (2) had at least two days of follow-up, with records of the clinical status, (3) did not deteriorate in the first two days of their follow-up, and (4) had at least two blood tests taken more than 12 h apart over the 12 days preceding one prediction event, or more. Patients who had died with no record of deterioration were excluded.
- Inclusion criteria set 3: For the external validation, the patients from Barzilai were included if they (1) had records of at least two days of follow-up before one of the deterioration-defining events has occurred, i.e., steroid therapy and mechanical ventilation (see below), (2) had at least two blood test records more than 12 h apart, over the 12 days preceding at least one prediction event time. Patients who had died with no record of the deterioration-defining events were excluded.

### Data processing

In the EHRs of the Sheba cohort, the clinical status evaluation was available, based upon internationally accepted guidelines, as described above. This evaluation, on a five-level scale, was defined as follows. Asymptomatic Infection: individuals who test positive for SARS-CoV-2 using a nucleic acid amplification test, but who have no relevant symptoms; Mild Illness: individuals who have any signs and symptoms of COVID-19 (e.g., fever, cough, sore throat, etc.) but without shortness of breath or abnormal chest imaging; Moderate Illness: individuals who show evidence of lower respiratory disease, still having an oxygen saturation (SpO_2_) ≥ 94% on room air; Severe Illness: individuals who have SpO_2_ < 94%, breaths/min ≥ 30, or lung infiltrates greater than 50%; and Critical Illness: patients with respiratory failure, septic shock, or multiple organ dysfunction. All the evaluations of the patients in the dataset were symptomatic, ranging from mild to critical. These data were converted into a binary representation, with the two better levels (mild and moderate) labeled as *non-severe* and the two worse levels as *severe.* In the Barzilai cohort, no clinical status evaluations were available. The records of the medical procedures were extracted for evaluating the time of the patient's deterioration (see below).

### Construction of training and validation datasets

Two alternative prediction tasks were considered for developing the dynamic predictive models. One task was to predict whether a currently *non-severe* patient will deteriorate (i.e., become *severe*) in the next two days (48 h). The other task was to predict whether the *non-severe* patient will deteriorate in the next four days (96 h). Accordingly, two prediction scopes were defined, one spanning the next 48 h and the other spanning the time interval of the next 96 h. The shorter scope covers the typical time of the subsequent status evaluation, and the longer one covers the next three status evaluations. A 3-h margin was added to both prediction scopes, to allow the inclusion of borderline cases.

A follow-up period was defined for each patient, from the first evaluation as *non-severe* until either deterioration (first evaluation as *severe*) or the last *non-severe* status evaluation if the patient did not deteriorate. For each patient, a sequence of events at which one could make the predictions was defined. For each event, the related dynamic features were computed from the blood parameter values, measured over the 12 days preceding this event, to serve as an input for the model. The outcome was defined based on the clinical status evaluation in the prediction scope of the event. The prediction events for the Sheba cohort were timed to every clinical status evaluation as *non-severe* during the follow-up. For each prediction event in the Sheba cohort dataset, the outcome was defined as equal to 1 or 0, according to whether the patient became *severe* within its prediction scope (i.e., during 48 or 96 h after the event). Events with no status evaluations within the prediction scope were omitted. The two resulting datasets (one for each prediction scope) were randomly split into 70% training and 30% testing, ensuring that all the events of the same patient are either entirely in training or in the testing subset to prevent information leakage.

For the Barzilai cohort, the prediction events were timed at 8 pm on each hospitalization day, starting 48 h after the admission. Since the Barzilai cohort records did not include the clinical statuses of the patients, the patient’s deterioration time was evaluated as the first initiation of mechanical ventilation or the first application time of steroids (Dexamethasone, Florinef, Hydrocortisone, Prednisone, Medrol, or Dexa-cortisone). The prediction events of this cohort were collected into two datasets, which differed only in the prediction scope used for outcome definition (48 h or 96 h). The event's outcome was defined by the patient's deterioration within its prediction scope (1) or else (0).

### Dynamic features and imputation of missing data

Dynamic features that captured each blood parameter's change over time were created in all the datasets. These features were constructed separately for each of the eight blood parameters and were used as an input for the prediction model, at each event. The dynamic features, constructed at each prediction event independently, comprised linear slopes of interpolated values over past time intervals of various durations and the average value of the parameter over the last 48 h. Missing average values were imputed by the median, separately for each blood parameter. Missing slope values for all blood parameters, except D-dimer, were imputed by the slope values from the larger intervals of the same parameter on the same event. Missing slopes for D-dimer were imputed using iterative imputation via the *IterativeImputer* function in Python. Events with entirely missing dynamic features of at least one parameter were excluded.

### Model training and validation

Five ML models were trained, including XGBoost, RandomForest, Light Generalized Boosting Machine, Support Vector Classifier, and regularized Logistic Regression, using *the scikit-learn* module v0.24.2 in Python 3.7. Each model was trained on the training sets (from the Sheba cohort) by an exhaustive search over a grid of meta-parameters, using the *GridSearchCV* function, with five-fold patient-grouped CV, with an objective of maximizing ROC AUC (see details in Supplementary Information, Table S1). The selection of the dynamic features was also altered during the training, using four, five, or seven of the slopes for each blood parameter, computed over various time intervals preceding the event. Different class weighting schemes were considered, including enforcing balanced weights. For every setup, defined by the choice of the model, the class weights, and the included dynamic features, the meta-parameters of the model were selected, which yielded the maximal ROC AUC. Then, the performance of all the selected models in all the setups was re-evaluated by the LOpO CV on the training set.

The dynamic features in the final model included slopes over the last two and seven days, and past intervals of days 4–2, 6–3, and 8–4 before the event. To calibrate the model's classification performance to a higher sensitivity level, the classification threshold of the final model was further tuned by fivefold CV on the training set for the objective of maximal specificity while maintaining sensitivity larger than 60%. The final model was tested on the internal and the external validation subsets (Table 1), constructing ROC curves, and evaluating ROC AUC, sensitivity, and specificity, with the tuned threshold values and a threshold of 0.5. All the above analysis, was performed separately for the time scopes of 48 h and 96 h, obtaining separate models and accuracy evaluations for the two time scopes.

### Ethics declaration

The Institutional Review Board of the Sheba Medical Center approved the study; Helsinki Committee approval Sheba Medical Center number—20-7953 and Barzilai Medical Center approval number 0003-21.

### TRIPOD guidance

The study was conveyed under transparent reporting of a multivariable prediction model for individual prognosis or diagnosis (TRIPOD) guidance^34^.

## Supplementary Information

Supplementary Information.

## Data Availability

No data repository is available for this study. Requests for the complete de-identified patient dataset addressed to the corresponding author will need to be reviewed by the Data Protection Officer of Sheba Medical Center and Barzilai University Medical Center. The authors made the appropriate materials available to the editorial staff during the review process to verify the results.
