# Supplementary material for: Developing and validating a machine learning prognostic model for alerting to imminent deterioration of hospitalized patients with COVID-19
Source: Sci Rep. 2022 Nov 10;12:19220. doi: 10.1038/s41598-022-23553-7 (PMC9648491; doi:10.1038/s41598-022-23553-7)
Supplement: Supplementary file 1 — Supplementary Information. [file 41598_2022_23553_MOESM1_ESM.pdf]

Supplementary information

Developing and Validating a Machine Learning Prognostic Model for Alerting to Imminent Deterioration of Hospitalized Patients with Covid-19

Kogan Yuri,*[1] Robinson Ari[1], Itelman Edward,[2] Bar-Nur Yeonatan[3], Jakobson Daniel Jorge[3,4], Segal Gad[2], Agur Zvia[1]

Institutional affiliation:

[1]Institute for Medical Biomathematics (IMBM), Israel.

[2]Department of Internal Medicine I, Chaim Sheba Medical Center, Sackler Faculty of Medicine, Tel Aviv University, Tel Aviv, Israel.

[3]Intensive Care Unit, Barzilai University Medical Center, Ashkelon, Israel.

[4]Faculty of Health Sciences, Ben-Gurion University of the Negev, Beer-Sheba, Israel

Supplementary figure S1:

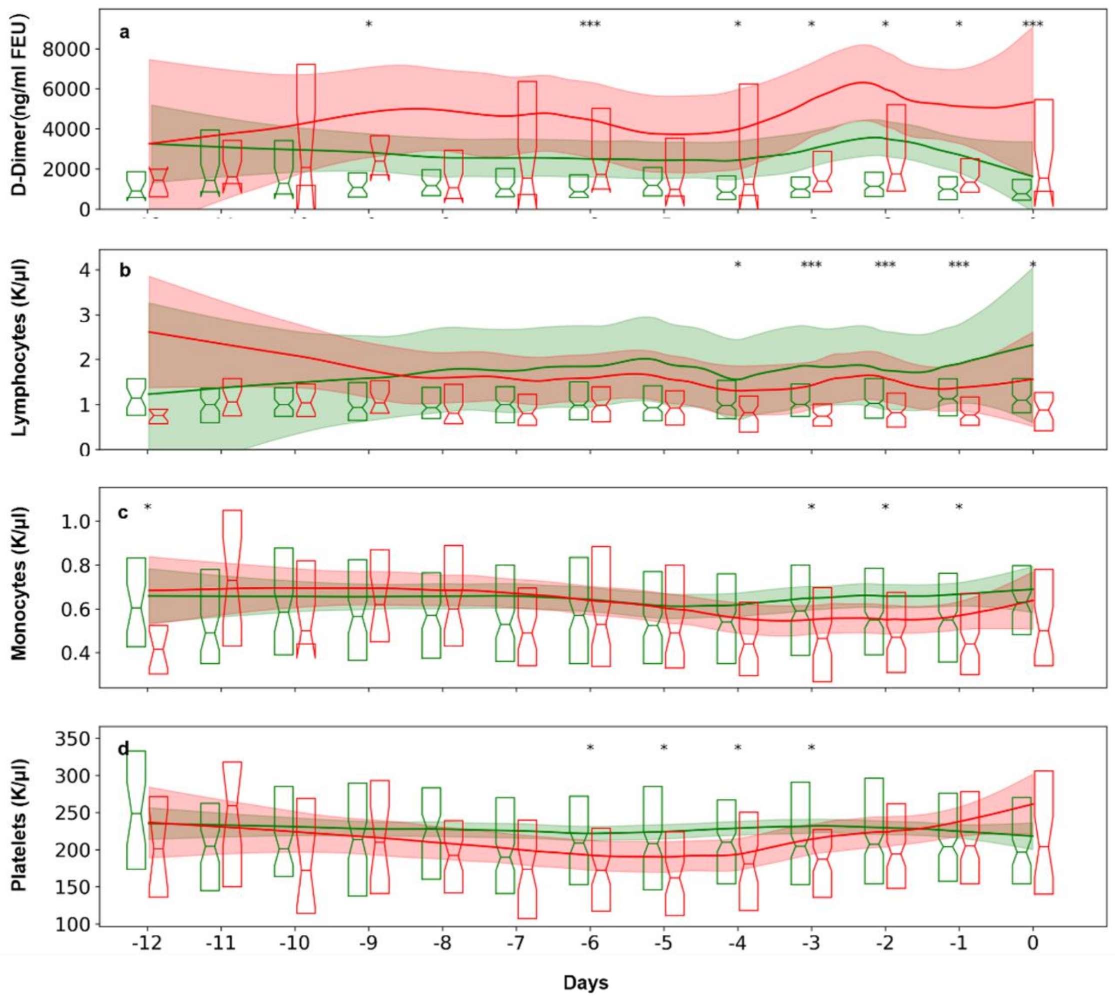

Figure S1. **Longitudinal measurements of less significantly diverting blood parameters in *non-severe* patients.** COVID-19 patients were aligned to t=0 by the day of their first deterioration from a *non-severe* to *severe* status (red), or by the end of follow-up time for patients who did not deteriorate (green). Summary boxplots show 25th, 50th, and 75th percentiles of pooled daily (a) D-Dimer values of 331 continually *non-severe* and 143 potentially *severe* patients, (b) Lymphocytes values of 595 *non-severe* and 208 *severe* patients, (c) Monocytes counts of 595 *non-severe* and 208 *severe* patients, (d) Platelets values of 593 *non-severe* and 208 *severe* patients. Locally Weighted Scatterplot Smoothing (Lowess), with 95% confidence intervals are shown in continuous lines and light bands around them. Asterisks represent the level of significance of the differences between the daily status groups, determined by the p-values of the Kruskall-Wallis test, *<0.05, **<0.01, ***<0.001.

Supplementary figure S2:

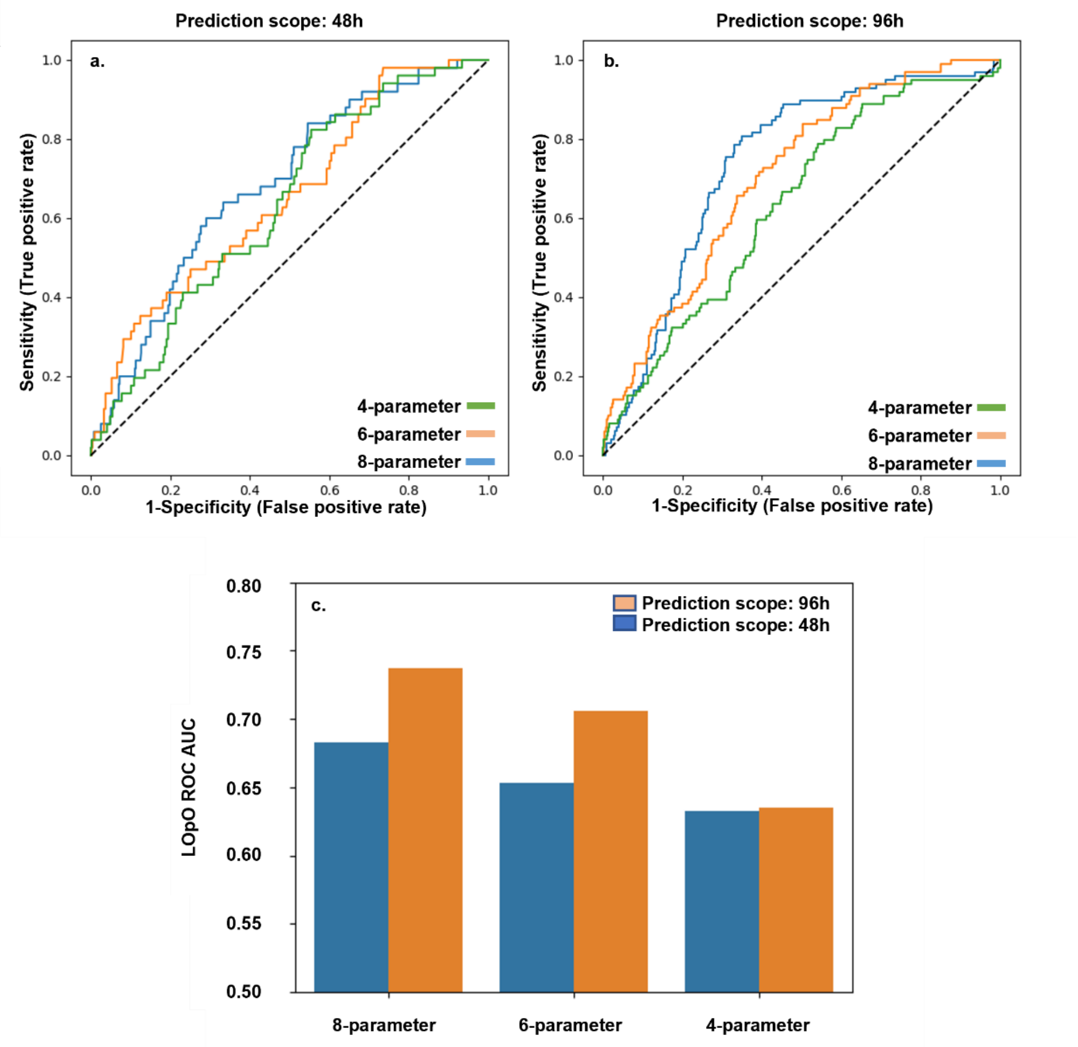

Figure S2. **Reduction of the number of input blood parameters.** LOpO CV ROC curves on the training set of the 8-parameter algorithm (CRP, LDH, D-Dimer, neutrophils, NLR, platelets, lymphocytes, monocytes), the 6-parameter algorithm (omitting D-Dimer and platelets) and the 4-parameter algorithm (omitting D-Dimer, platelets, lymphocytes, monocytes), with prediction scope of 48 hours (a) and 96 hours (b). The values of the ROC AUC for the 8-parameter, 6-parameter and 4-parameter algorithms shown in a bar plot, for time scopes of 48 hours (blue, AUC=0.68,0.65,0.63 respectively) and 96 hours (orange, AUC=0.73,0.70,0.63 respectively) (c).

Supplementary table S3:

Model	Meta-parameter	Values
**Logistic Regression with ‘elasticnet’ penalty**	C (regularization strength)	11 points evenly spaced on a log scale between 10^{-4} and 1
	l1_ratio (Elastic-Net mixing parameter)	37 point spaced between 0 and 1
	class_weight (weight of 1 relative to 0)	balanced, 1, 3, 5
**SVC with ‘rbf’ kernel**	C (regularization parameter)	10 points evenly spaced on a log scale between 10^{-2} and $10^{0.5}$
	gamma (kernel coefficient)	8 points evenly spaced on a log scale between 10^{-4} and 1
	class_weight (weight of 1 relative to 0)	balanced, 1, 3, 5
**RandomForest, with 100 trees as base estimators**	max_depth (maximal tree depth)	4, 8, 12
	min_samples_leaf (minimal size of a leaf at split)	4, 8, 24, 48
	max_features (proportion of features sampled for each base estimator)	0.5, 0.75, 1
	max_samples (proportion of points sampled for each base estimator)	0.67, 0.85, 1
	class_weight (weight of 1 relative to 0)	balanced, 1, 3, 5
**XGBoost, with 200 trees as base learners**	max_depth (maximal tree depth)	4, 6, 8
	learning_rate	0.001, 0.005, 0.01, 0.03, 0.05, 0.08, .1
	min_child_weight (minimal size of a leaf at split)	4, 12, 24, 48
	colsample_bytree (proportion of features sampled for each base estimator)	0.4, 0.7, 0.9
	subsample (proportion of points sampled for each base estimator)	0.5, 0.75, 1
	scale_pos_weight (weight of 1 relative to 0)	balanced, 1, 3, 5
**LightGBM, with 100 trees as base estimators**	num_leaves (maximal number of leaves per tree)	31, 80
	max_depth (maximal tree depth)	-1, 2, 4
	learning_rate	0.01, 0.05, 0 .1, 0.5
	min_child_samples (minimal size of a leaf at split)	10, 20, 40
	colsample_bytree (proportion of features sampled for each base estimator)	0.5, 0.7
	subsample (proportion of points sampled for each base estimator)	0.5, 0.9
	class_weight (weight of 1 relative to 0)	balanced, 1, 3, 5

Table S3. **Listing of meta-parameter grid values for the trained models.** For each model we describe the specifics of its realization (first column), the list of the meta-parameters, changed during the tuning

(second column), and the values used for the tuning (third column). The grid was formed by all the possible combinations of meta-parameter values, and the tuning of each model consisted of an exhaustive search over its grid. We also trained each model over the same grid, fixing the class weights at balanced values. At each point, the performance metrics was evaluated by 5-fold cross-validation.
